# An Unusual Cause of Small Bowel Obstruction: A Case Report

**DOI:** 10.7759/cureus.1116

**Published:** 2017-03-26

**Authors:** Sundaramurthi Sudharsanan, TP Elamurugan, Chellappa Vijayakumar, Kumar Rajnish, Sadasivan Jagdish

**Affiliations:** 1 Surgery, Jawaharlal Institute of Postgraduate Medical Education and Research (JIPMER), Puducherry, India.

**Keywords:** intestinal obstruction, enterolith, surgical emergency, enterotomy

## Abstract

Small bowel obstruction is a common surgical emergency. The common causes are adhesions, malignancies, and hernias. We present a rare case of small intestinal obstruction caused by an enterolith in the distal ileum in a patient with an apparently normal gut.

A 59-year-old male who underwent gastrojejunostomy 15 years back presented with features of intestinal obstruction of five days' duration. After initial conservative management, the patient was taken up for laparotomy. An enterolith causing obstruction was found in the distal ileum, and it was crushed and milked into the colon. The patient made an uneventful recovery.

The chyme crossing the ileum is usually liquid or semi-solid and hence luminal obstruction by the faecal bolus in the ileum is very unusual. In patients with previous gastric surgeries where the pylorus is bypassed, the solid food particles enter the small intestine and can form a bezoar. This patient was managed with laparotomy and milking of the stool bolus into the colon. Other treatment options include enterotomy or resection of the diseased bowel and removal of the enterolith.

Small bowel obstruction due to an enterolith is very rare and can pose a diagnostic challenge.

## Introduction

Small bowel obstruction is one of the common surgical emergencies we see in our day-to-day practice. Postoperative adhesions (60%) are the most common cause of small bowel obstruction, followed by malignant deposits or extrinsic compression of the small bowel by other primary malignancies (20%) and intestinal hernias (10%) [1]. Intraluminal causes of small bowel obstruction in adults are rare. Here, we report a rare case of small intestinal obstruction caused by an intraluminal enterolith.

## Case presentation

**Figure 1:**
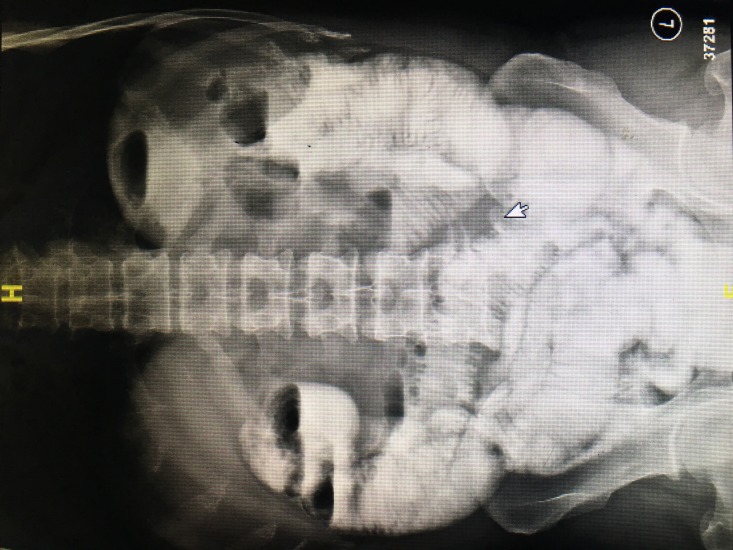
Oral contrast study showing multiple air-fluid levels

A 59-year-old male presented to the emergency department with complaints of diffuse abdominal pain, vomiting, and abdominal distension for five days. He reported not passing stools for two days. He had an elective laparotomy done 15 years back for peptic ulcer disease in our institute, the details of which were not known. On examination, he was conscious, oriented, and his vitals were stable. His abdomen was soft and distended with exaggerated bowel sounds. On digital rectal examination, the rectum was empty. Plain x-ray of the abdomen did not show any evidence of acute intestinal obstruction. An ultrasound of the abdomen was done which showed dilated hyper-peristaltic small bowel loops of maximum caliber 3.5 cm. There was no free-fluid in the peritoneal cavity and the gall bladder was normal. A clinical diagnosis of the adhesive intestinal obstruction was made, and the patient was managed conservatively with intravenous fluids and nasogastric drainage. Following 48 hours of conservative treatment, the patient had clinical improvement and was started on oral fluids. After two days of starting oral feeds, the patient again developed abdominal distension with bilious vomiting. A water-soluble contrast study was done which revealed dilated jejunal loops with multiple air-fluid levels and slow filling of the contrast till the ileum [Figure 1].

**Figure 2:**
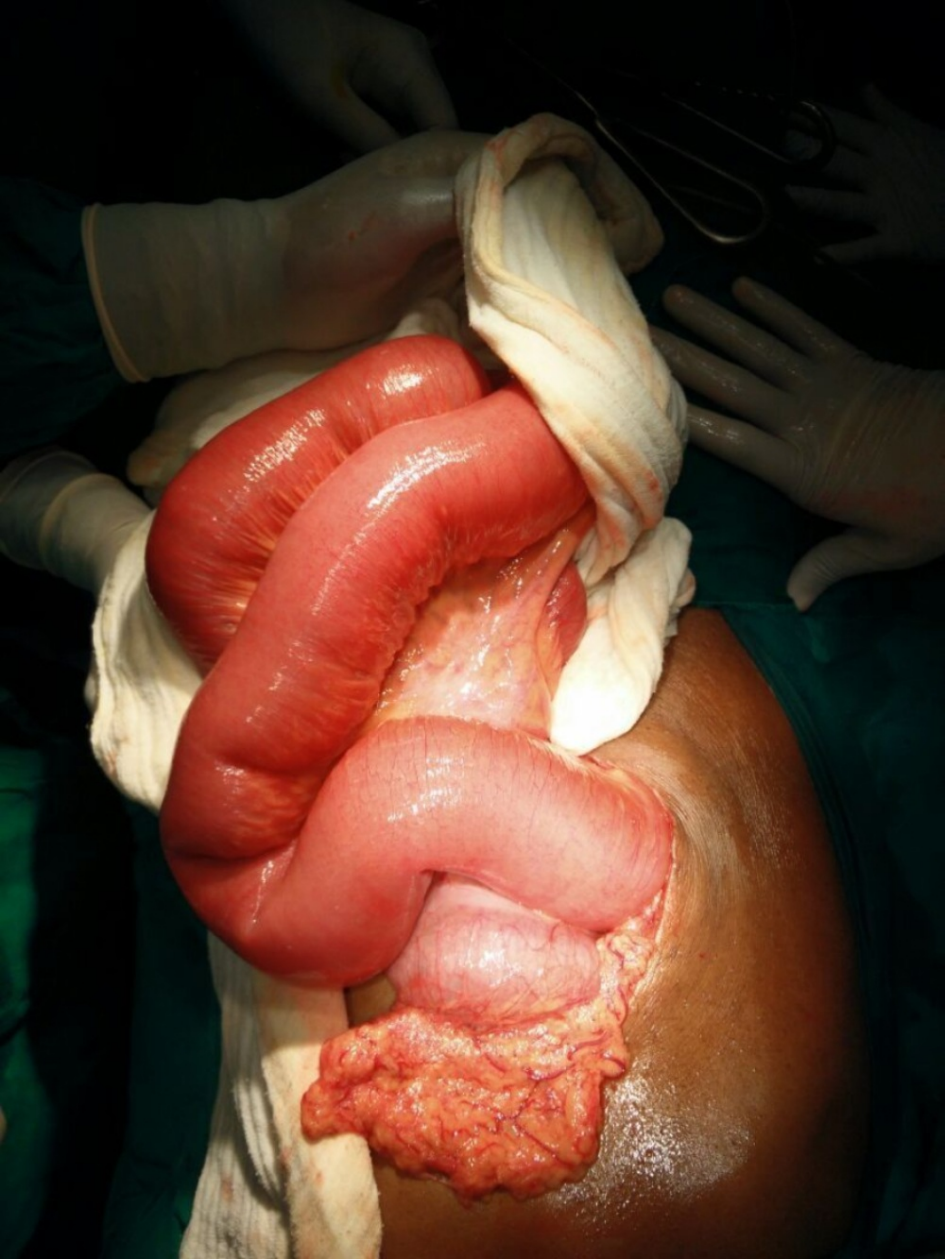
Dilated jejunal bowel loops on laparotomy

**Figure 3:**
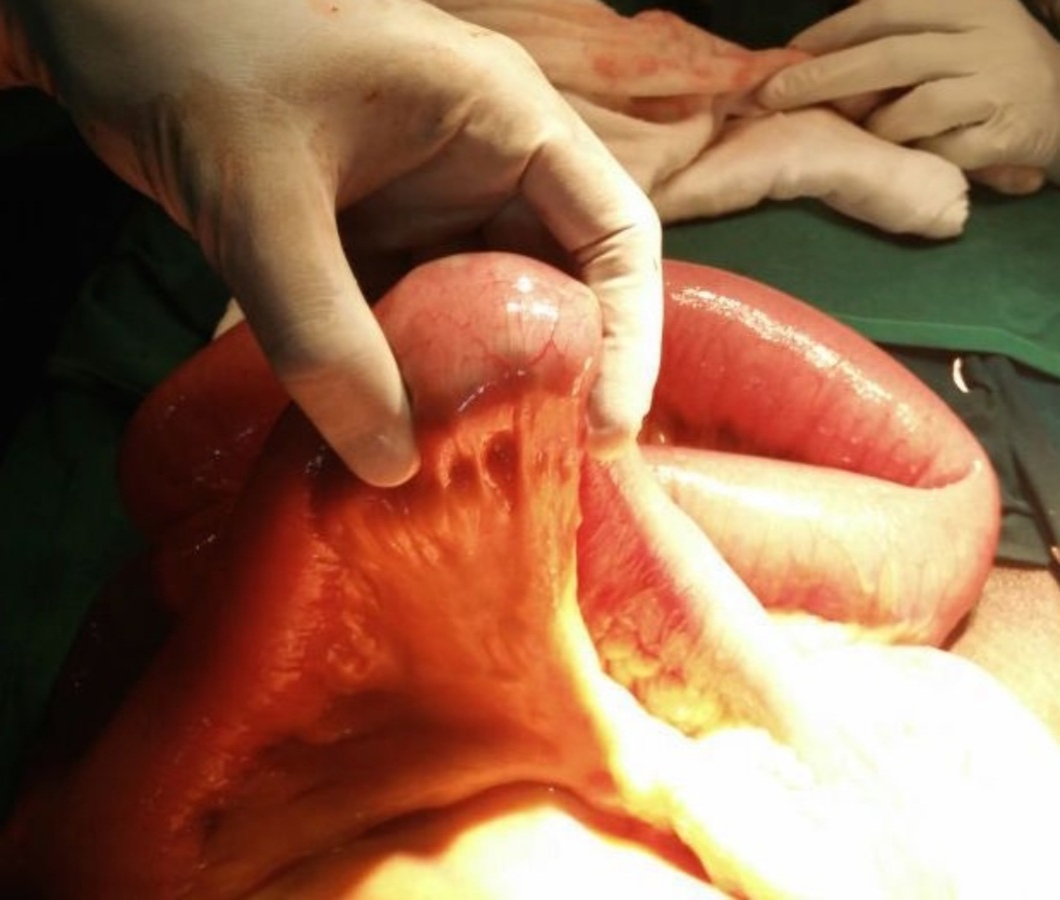
Intraluminal enterolith causing the intestinal obstruction

In view of the persistent high nasogastric drainage of more than one litre a day and diffuse abdominal pain with guarding, the patient was planned for an exploratory laparotomy. Intraoperatively, there were no adhesions. A 6 × 5 cm well-defined firm intraluminal mass was palpable in the ileum about 45 cm proximal to the ileocaecal junction. The bowel loops proximal to the mass were dilated and the loops distal to it were collapsed [Figures 2-3]. The mass, which was firm but mouldable with pressure, was found impacted at the transition area in the bowel. There was no structural abnormality identified in the bowel or the gall bladder. The mass, probably a stool bolus or fecal mass, was crushed and milked into the caecum.

The postoperative period was uneventful. The patient was started on oral feeds on postoperative day two and was discharged on postoperative day six. On follow-up, an oral contrast study was done, which showed normal study of the bowel.

## Discussion

Enteroliths are abnormal concretions occurring in the intestine and are usually composed of mineral salts [2]. An enterolith was first described by a French physician, Chomelin J in 1710, as a case of stone formation in a duodenal diverticulum at autopsy [3]. Sjoqvist gave the chemical composition of an enterolith in 1908 [4].The prevalence of enteroliths is reported to be 0.3% to 10% in various populations [5]. The exact incidence of enterolithiasis is not known because most cases are asymptomatic or they are not diagnosed or they remain underreported.

Enteroliths are classified as primary enteroliths (that form within the bowel) or secondary enteroliths (that form outside the bowel and migrate to the intestine). Gall stones and renal calculi can erode into the adjacent bowel, resulting in secondary enteroliths.

They are also classified as true or false enteroliths based on their chemical composition. True enteroliths are composed of substances normally found in the gut. Choleic acid stones, calcium stones, ammonium-magnesium phosphate stones, and hydroxy-fatty acid stones are examples of true enteroliths. False enteroliths are usually formed by substances that are not normally found in the gut. They include undigested bezoars, precipitation of insoluble varnish stones, concretions of chalk, lime or barium sulfate stones, and inspissated faecal stones.

The most common factor involved in the development of an enterolith is stasis within the bowel as a result of altered endoluminal propagation and peristaltic functionality. Stasis occurs frequently at areas of intestinal diverticula, sites of intestinal anastomoses, Roux-en-Y sites, intestinal kinking (adhesions), incarcerated hernias, and at sites of intestinal strictures [5]. The microenvironment and luminal pH specific to each segment of the gut with varying digestive properties can result in crystallisation of different substances at different sites of the small intestine. Choleic acid aggregates and forms enteroliths at acidic pH whereas calcium enteroliths are formed at alkaline pH. Hence cholic acid enteroliths are seen in the proximal small intestine and calcium containing enteroliths are almost always seen in the distal ileum or colon [6]. Altered anatomy (as in the case of gastrojejunostomy), where the pylorus is bypassed and solid food directly enters the small intestine, can promote the development of an enterolith. In the present case, the patient had a gastrojejunostomy, which could have been one of the factors that led to the development of the enterolith. The pylorus controls the flow of chyme from the stomach to the duodenum. 

The presentation is usually non-specific with most enteroliths remaining asymptomatic. The enterolith tumbling through the bowel lumen can result in fluctuating subacute type of intestinal obstruction. They can also present as surgical emergencies like acute intestinal obstruction, hemorrhage, and bowel perforations. In the present case, the patient presented with features of acute intestinal obstruction due to the impacted faecal mass in the ileum that warranted a surgical management. 

An abdominal x-ray is usually the first investigation in a patient who presents with features of intestinal obstruction. Only one-third of the enteroliths are radio-opaque [7]. Radio-opaque stones have peripheral calcifications with a radiolucent centre and are usually seen in the right iliac fossa region in a roentgenogram. Apparent mobility of the stone in serial radiological examinations strongly suggests the presence of an intraluminal enterolith. The use of computed tomography (CT) scan with oral contrast can increase the yield of diagnosing an enterolith. A CT scan can also identify radiolucent enteroliths, the exact location, and the total number of enteroliths along with the presence of underlying bowel pathology. In most cases, the diagnosis of enterolithiasis could be made only at the time of laparotomy. 

In enteroliths less than 2 cm in size and without any luminal compromise, conservative management with serial abdominal examinations, nil per oral, and intravenous fluids along with nasogastric suctioning can be attempted. Most enteroliths pass spontaneously with the above management [8]. Endoscopic retrieval of enteroliths can be done if they are located in the duodenum. Larger stones can be managed by endoscopic electrohydraulic lithotripsy (EEHL) and endoscopic mechanical lithotripsy (EML). Single and double balloon enteroscopy with carbon dioxide insufflation have also been described for management of enteroliths in the proximal small intestine [9].

Surgical management is reserved in cases of large stones when interventional methods are not available and when expectant management fails. Digital fragmentation of the stone followed by manual milking of the smaller parts into the large intestine is successful in 50% of the cases [10]. Hard enteroliths that cannot be fragmented mechanically can be retrieved by a proximal enterotomy with manual enterolith removal. Segmental small bowel resection with primary anastomosis will be needed in cases of enteroliths associated with bowel pathologies like diverticulum, stricture, etc. Surgical management of gall-stone ileus includes enterolithotomy along with cholecystectomy and closure of the fistula in a single stage or two-staged procedure.

## Conclusions

Most reported cases of enteroliths occur in pathologic bowels, as in diverticular diseases or inflammatory strictures. This is a case of an enterolith occurring in a normal small bowel, and the location of the enterolith (about 45 cm proximal to the ileocaecal junction without any local abnormalities at the site of impaction) makes this presentation a very rare one. One possible factor in this patient is the presence of a gastrojejunostomy, which can cause under-processed food in the stomach to enter the jejunum directly and go on to develop into an enterolith.
